# Silk fibroin microgels as a platform for cell microencapsulation

**DOI:** 10.1007/s10856-022-06706-y

**Published:** 2022-12-31

**Authors:** Nina Bono, Giulio Saroglia, Stefania Marcuzzo, Eleonora Giagnorio, Giuseppe Lauria, Elena Rosini, Luigi De Nardo, Athanassia Athanassiou, Gabriele Candiani, Giovanni Perotto

**Affiliations:** 1 Department of Chemistry, Materials and Chemical Engineering “Giulio Natta”, Politecnico di Milano, Via Mancinelli 7, 20131 Milan, Italy; 2 Smart Materials, Istituto Italiano di Tecnologia, Via Morego 30, 16163 Genova, Italy; 3 Neurology IV-Neuroimmunology and Neuromuscular Diseases Unit, Fondazione IRCCS Istituto Neurologico Carlo Besta, Via Celoria 11, 20133 Milan, Italy; 4 Department of Clinical Neurosciences, Fondazione IRCCS Istituto Neurologico Carlo Besta, Via Celoria 11, 20133 Milan, Italy; 5 Department of Medical Biotechnology and Translational Medicine, University of Milan, Via Vanvitelli 32, 20133 Milan, Italy; 6 The Protein Factory 2.0, Department of Biotechnology and Life Sciences, University of Insubria, Via J.H. Dunant 3, 21100 Varese, Italy

## Abstract

Cell microencapsulation has been utilized for years as a means of cell shielding from the external environment while facilitating the transport of gases, general metabolites, and secretory bioactive molecules at once. In this light, hydrogels may support the structural integrity and functionality of encapsulated biologics whereas ensuring cell viability and function and releasing potential therapeutic factors once in situ. In this work, we describe a straightforward strategy to fabricate silk fibroin (SF) microgels (µgels) and encapsulate cells into them. SF µgels (size ≈ 200 µm) were obtained through ultrasonication-induced gelation of SF in a water-oil emulsion phase. A thorough physicochemical (SEM analysis, and FT-IR) and mechanical (microindentation tests) characterization of SF µgels were carried out to assess their nanostructure, porosity, and stiffness. SF µgels were used to encapsulate and culture L929 and primary myoblasts. Interestingly, SF µgels showed a selective release of relatively small proteins (e.g., VEGF, molecular weight, M_W_ = 40 kDa) by the encapsulated primary myoblasts, while bigger (macro)molecules (M_W_ = 160 kDa) were hampered to diffusing through the µgels. This article provided the groundwork to expand the use of SF hydrogels into a versatile platform for encapsulating relevant cells able to release paracrine factors potentially regulating tissue and/or organ functions, thus promoting their regeneration.

**Graphical Abstract:**

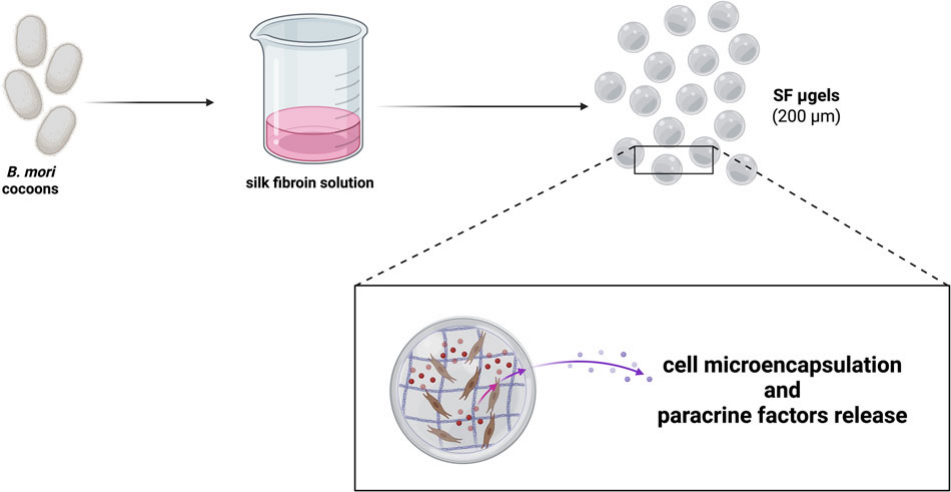

## Introduction

Cell microencapsulation is a very promising technique whereby living cells are entrapped within a biocompatible membrane in an attempt to insulate the (engrafted) cells from the host immune attack and enhance or prolong their function in vivo [1–8]. Several approaches have been devised to encapsulate various cell types and treat a wide range of diseases such as diabetes, cancer, central nervous system diseases, and endocrinological disorders [9–11]. Most of these strategies rely on the use of a permeable membrane that enables cells to be nourished while allowing catabolic waste and paracrine factors secreted by the encapsulated cells to exit the encapsulating device.

In the search for the most suitable biomaterial for cell microencapsulation, synthetic polymers (e.g., poly(ethylene glycol) (PEG) [12, 13], polyurethane [14], polyvinyl alcohol (PVA) [15]) have been extensively explored. Although microcapsules can be shaped with these materials through different methods, most microencapsulation systems rely on natural polymer-based hydrogels, such as alginate [16–18] and hyaluronic acid [19, 20], due to their high water content and consistency mimicking the physical features of the extracellular matrix (ECM) [21, 22]. For instance, alginate has been extensively employed in microencapsulation approaches because it is biocompatible, widely available, and easily cross-linked in the presence of divalent cations, such as Ca^2+^. Alginate creates dense hydrogels with high stiffness but low biodegradability [23, 24] and without cell adhesion motifs, making the cell-polymer interactions somewhat restrained. To overcome this issue, alginate has preferably been used in combination with other biopolymers [25]. Hydrogels made of fibrous protein-based hydrogels, such as collagen [26, 27] and keratin [28, 29], are also attractive because they are very similar to the native ECM, can be easily processed and functionalized in water under mild conditions, and possess consensus motifs that cells recognize and bind to [30, 31]. However, the poor mechanical properties make these materials hard to handle and process.

Silk fibroin (SF), the protein extracted from the *Bombyx mori* (*B. mori*) silkworm cocoon, possesses a unique combination of interesting features that turn it into a very appealing biomaterial. These peculiar properties arise from the well-ordered structure of the SF itself, which comprises a highly repetitive Gly-Ser-Gly-Ala-Gly-Ala (GSGAGA) motif interlaced by regions rich in polar amino acids [32, 33]. Such repetitions make SF able to self-assemble from its water solution into different crystalline structures [34–38]. The ease of processing, excellent biocompatibility, remarkable mechanical properties, and tailorable degradability of SF has been exploited for the fabrication of various articles such as films, porous matrices, nonwoven mats, to be used for wound healing, tissue engineering (i.e., bone, tendon, ligament, cartilage, skin, liver, trachea, nerve, cornea, eardrum, dental, bladder) and drug delivery [39–42]. Silk can be fabricated in a multitude of material formats and the development of material fabrication techniques drove the use of SF as a biomaterial, enabling more and more applications. When using hydrogels of SF, there is a current lack of methods to fabricate microcapsules. Indeed, in many cell-based applications, gelation must be induced under mild conditions in a relatively short time. Unfortunately, in the absence of chemical modifications or non-physiological treatments, such as the use of cross-linking agents and/or low pH (<5) and/or high temperature (>60 °C), the sol-gel transition from an aqueous solution of SF to a β-sheets-rich hydrogel [43] is a spontaneous yet inconveniently slow process. These shortcomings have dramatically restrained the use of SF hydrogels in biomedicine.

In this background, we propose a clever strategy based on a water-in-oil (W/O) emulsion to fabricate SF µgel capsules and embed living cells. Drawing inspiration from the work of Wang and co-authors, we employed ultrasonication to shorten the gelation time of SF from weeks to minutes [44–47]. The gelation kinetics of the SF µgels was optimized as a function of the ultrasonication duration, the degumming time, and the concentration of SF. The µgels were characterized to evaluate their structure, mechanical properties, permeability to biomacromolecules and to optimize the cell encapsulation efficiency. After the encapsulation of primary myoblasts, we evaluated cell viability and proliferation as well as the secretion and the release from the SF µgels of soluble factors with paracrine activity.

## Materials and methods

### Materials

*B. mori* silkworm cocoons were purchased from Cantiere della Provvidenza SPA Scs Onlus (Belluno, Italy). L929 cell line (NCTC clone 929, CCL-1™) was from the American Type Culture Collection (ATCC, Manassas, VA, USA). BCA Protein Assay Kit and Live/Dead Cell viability Assay were from ThermoFisher (Monza, Italy). *Escherichia coli (E. coli)* JM109 bacteria (Gram-negative bacterial strain, biosafety level: 1) were purchased from Leibniz Institute DSMZ- German Collection of Microorganisms and Cell Cultures (Braunschweig, Germany). All the other chemicals were from Merck Life Science (Milan, Italy), if not differently specified.

### Preparation of SF solutions

Solutions of SF at different molecular weights (M_W_) were prepared according to a protocol reported in the literature [48]. Briefly, 5 g of *B. mori* silk cocoons were boiled (degumming process) for 20 min, 30 min, or 40 min in 2 L of 0.02 M sodium carbonate, giving rise to 20, 30, and 40 min of boiling (MB) solutions, respectively [49]. Silk fibers were next rinsed three times in deionized water (dH_2_O), air dried at room temperature (r.t.), dissolved in a 9.3 M LiBr at 60 °C for 4 h, and dialyzed against MilliQ water for 3 days (two water changes per day). The resulting SF solutions (SF = 60 mg mL^−1^) were centrifuged to remove impurities. The SF solutions were stored at 4 °C until use.

### Determination of SF gelation time

To assess the gelation time, 5 mL of 20, 30, and 40 MB SF solutions were prepared at different protein concentrations (10, 12, 15, 18, and 20 mg mL^−1^) in MilliQ water, phosphate-buffered saline (PBS), or cell culture medium (Dulbecco’s Modified Eagle Medium, DMEM). The SF solutions were next sonicated at a frequency of 20 kHz and different amplitudes (20, 30, and 40%) for 5, 10, 15, and 20 s using a Fisherbrand™ Model 505 Sonic Dismembrator (Fisher Scientific Italia, Rodano, Italy) equipped with a 13 mm diameter-tapered microtip. The solutions were kept at 37 °C after sonication and the sol-gel transition was visually monitored.

### Preparation and characterization of cell-free SF µgels

#### Preparation of cell-free SF µgels

For the preparation of SF µgels (Fig. S1), 5 mL of sonicated 30 MB SF solution was transferred in a 10 mL syringe equipped with a 20 G needle and connected to a Tygon^®^ tube (inner ∅: 1 mm, length: 150 mm). The syringe was then placed into the bed of a syringe pump (Landgraf LA-100, Colaver, Vimodrone, Italy) set at 250 µL min^−1^ to allow dripping of the SF solution onto the 2% (v/v) Span80-in-oil emulsion (batch emulsion procedure). The emulsion was stirred (2000 rpm) for 1 h at 37 °C, then washed three times with PBS to separate the µgels from the oil phase. The SF µgels were then resuspended in PBS and further analyzed.

#### Morphological and physicochemical characterization of cell-free SF µgels

The nanostructure of SF µgels was examined with a cold field emission Scanning Electron Microscope (SEM Jeol JSM-7500FA, Tokyo, Japan). Briefly, SF µgels were dehydrated in graded ethanol series (50, 70, 90, 95, and 100%), then transferred into a critical point dryer (CPD, K850WM, Quorum Technologies, Lewes, UK) and rinsed 5 times with CO_2_ under supercritical conditions. Samples were mounted onto conductive carbon tape, gold-sputtered, and examined using an accelerating voltage of 10 kV. Fiber and pore dimensions were quantified on SEM images using ImageJ (https://imagej.nih.gov/ij/).

The chemical structure of SF gels was assessed by Fourier Transformed Infrared Spectroscopy (FTIR) performed on a Vertex 70 v FTIR (Bruker, MA, USA) equipped with a MIRacleATR accessory (PIKE Technologies, WI, USA). Spectra were measured in the range from 400 cm^−1^ to 4000 cm^−1^ with a resolution of 4 cm^−1^ and a 64 scan-acquisition. Quantification of SF secondary structure (β-sheets content) was performed by the self-deconvolution of the Amide I region according to the literature [35, 38].

#### Mechanical characterization of cell-free SF µgels: microindentation tests

Mechanical properties of SF gels, fabricated as both µgels and macroscopic cylindrical samples (∅: 10 mm, height: 16 mm, prepared as described in Supplementary Information S1), were characterized by nanoindentation tests using a Chiaro Nanoindenter (Optics11 Life, Amsterdam, NL) equipped with a SiO_2_ bead tip (size: 26 µm) and a cantilever with an elastic constant of 0.480 N m^−1^.

Briefly, SF gels prepared at different SF concentrations (10, 12, 15, 18, and 20 mg mL^−1^; *n* ≥ 6 per condition), were transferred into a glass Petri dish and soaked in PBS (Fig. S2A-B). To compare the mechanical properties of SF µgels and their corresponding macroscopic counterparts, we tested the surface and the core of macro-sized hydrogels by microindentation (please refer to Supplementary Information S1).

For both µgels and macroscopic hydrogels, microindentation tests were performed at 800 nm s^−1^ with an indentation depth of 2.5 µm. The compressive (elastic) modulus was determined by an automated Hertzian best fit of the linear part of the indentation curve (Fig. S2C-D).

### Evaluation of the sterilization of the SF solution by ultrasonication

*E. coli* bacteria were pre-cultured in 5 mL of Luria-Bertani broth at 37 °C under shaking at 130 rpm for 20 h, until reaching an optical density at λ = 600 nm (OD_600nm_) of ≈1, corresponding to ≈10^9^ bacteria mL^−1^. Bacterial suspension was next mixed with 4 mL of 30 MB SF in DMEM to obtain a final SF concentration of 15 mg mL^−1^ and a bacterial concentration of ≈10^7 ^mL^−1^. The SF-bacteria mixture was then sonicated for 10 s at 20 kHz and 30% amplitude. Afterward, the number of viable bacteria was determined using the pour plate method. Briefly, the number of colony-forming units (CFUs) was counted on LB-agar Petri dishes where serial 10-fold serially diluted bacteria were plated and incubated for 24 h at 37 °C. The SF-bacteria mixture not subjected to ultrasonication was used as the positive control for bacterial viability.

### Cell encapsulation in SF µgels

#### Cell culture

Mycoplasma-free L929 cells were expanded in T75 flasks in high glucose DMEM supplemented with 1 mM sodium pyruvate, 10 mM HEPES buffer, 100 U mL^−1^ penicillin, 0.1 mg mL^−1^ streptomycin, 2 mM glutamine and 10% (v/v) fetal bovine serum (FBS) (hereafter referred to as complete DMEM, cDMEM), in a humidified atmosphere and 5% CO_2_ at 37 °C (hereafter referred to as standard culture conditions). For encapsulation, the cells were detached with a 1× trypsin-EDTA solution and resuspended in cDMEM before use.

#### Culture and biochemical characterization of L929 cells-laden SF µgels

**Fig. 1:**
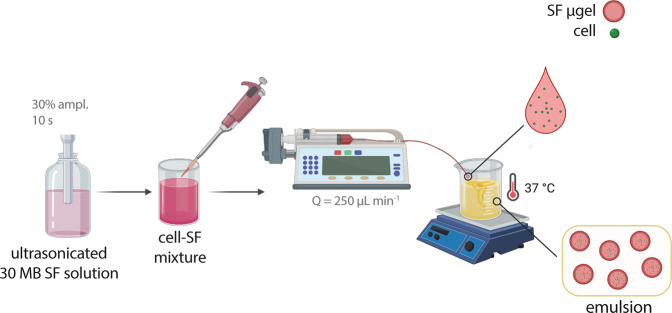
Schematic for the production of cells-SF µgels by water-oil emulsion procedure. Image created with BioRender.com

L929 cells-laden SF µgels were prepared by batch emulsion procedure as depicted in Fig. 1. Briefly, L929 cell suspension was mixed 1:4 (v/v) with 30 MB SF solution in DMEM to give a final cell concentration of 2.5 × 10^7^ cells per mL and SF concentration of 15 mg mL^−1^. Next, 5 mL of the cell-SF mixture were loaded into a 10 mL syringe and dripped onto the Span80-in-oil emulsion at a flow rate of 250 µL min^−1^ (batch emulsion procedure). The emulsion was stirred for 1 h at 37 °C until gelation occurred, then the cells-µgels were washed in PBS, collected, resuspended in cDMEM, and incubated in standard culture conditions for different periods (1, 4, and 7 days).

The cell viability was assessed at different time points by the Live/Dead Cell Viability assay. Briefly, L929 cell-laden µgels were rinsed with PBS and incubated with 1 µM calcein AM and 2.5 µM propidium iodide to stain live (green) and dead (red) cells, respectively. After a 15-min incubation at 37 °C in the dark, the samples were washed with PBS. Fresh culture medium was added before imaging under the fluorescence microscope (calcein AM: λ_em_ = 494 nm, λ_em_ = 517 nm; propidium iodide: λ_em_ = 536 nm, λ_em_ = 617 nm; 40× magnification).

To evaluate the proliferation of the encapsulated cells over time, the total DNA content was quantified. Briefly, L929 cell-laden SF µgels were harvested at different time steps (1, 4, and 7 days post-cell encapsulation), washed in PBS, and resuspended in 200 µL of homogenization buffer (0.01% (v/v) Triton-X in 50 mM Hepes). Following 3 freeze-thawing cycles, the samples were disrupted with a tissue homogenizer and centrifuged at 12,000 rpm for 5 min at 4 °C. Afterward, 20 µL of supernatant were mixed with 10 µL of 200× SYBR Green I (λ_ex_ = 497 nm, λ_em_ = 520 nm), incubated for 15 min in the dark and the fluorescence was read with a Synergy H1 spectrophotometer (BioTek Instruments, Milan, Italy) (*n* = 3 samples per condition). Salmon sperm DNA (0–2 µg mL^−1^) was used to construct a standard curve for DNA quantifications. The DNA content of each sample was normalized to the total protein content, as determined by the BCA assay. Data were expressed as µg of DNA per mg of SF.

### Microencapsulation with primary myoblasts

#### Animals

Transgenic SOD1-G93A (male) mice (B6SJL-Tg(SOD1*G93A)1Gur) were purchased from Charles River, Inc. (Wilmington, MA, USA), maintained, and bred at the animal house of the Fondazione IRCCS Istituto Neurologico “Carlo Besta” in compliance with institutional guidelines, international regulations (EEC Council Directive 86/609) and project approvals from the Italian Ministry of Health (ref. IMP183/2018-PR). SOD1-G93A mice were euthanized at week 18 with CO_2_.

#### Primary myoblasts isolation and culture

Gastrocnemius muscle biopsies were obtained from G93A-SOD1 mice at week 18 [50]. Finely cut muscle fragments were seeded onto 60 mm dishes and cultured in high glucose DMEM supplemented with 100 U mL^−1^ penicillin, 0.1 mg mL^−1^ streptomycin, 2 mM glutamine, 20% (v/v) FBS, 10 μg mL^−1^ insulin, 2.5 ng mL^−1^ basic fibroblast growth factor (bFGF; Prepotech, London, UK), and 10 ng mL^−1^ epidermal growth factor (EGF; Prepotech). After 10–15 days of culture, the confluent monolayers were detached and sub-cultured. The purification of primary myoblasts was performed through pre-plating treatment. The cells were tested against mycoplasma contamination before use.

#### Myoblasts encapsulation

Myoblasts-encapsulating SF µgels were prepared as described herein above (Fig. 1). Briefly, 10 s sonicated SF solution (in DMEM) was mixed with myoblast cell suspensions to give a cell-SF mixture (1:4 (v/v)) at a final cell concentration of 2.5 × 10^6^ cells per mL and SF concentration of 15 mg mL^−1^. Next, 5 mL of the cell-SF mixture were dripped into the Span80-in-oil emulsion at a flow rate of 250 µL min^−1^ (batch emulsion procedure). The emulsion was stirred for 1 h at 37 °C until gelation, then the cells-µgels were washed in PBS, collected, resuspended in cDMEM, and incubated in standard culture conditions for different periods of time (1, 4, and 7 days). At any time point, 1 mL of the cell culture medium containing the myoblasts-SF µgels was collected, then the µgels and supernatant were separated by gentle centrifugation and processed for further analyses. Experiments were performed at least in triplicate.

#### Gene expression in encapsulated myoblasts

The total RNA from myoblasts-SF µgels was extracted using TRIzol (Thermo Fisher Scientific), according to the manufacturer’s instruction. The RNA quality was verified using a 2100 Nano Bioanalyzer (Agilent Technologies, Waldbronn, Germany). Total RNA was then retrotranscribed using the SuperScript Vilo cDNA Synthesis kit (Thermo Fisher Scientific) in a thermocycler system, according to the following thermal protocol: 25 °C for 10 min, 42 °C for 60 min, 85 °C for 5 min. Afterward, quantitative Real-Time (q-RT) PCR reactions were carried out in a ViiA7 RT-PCR System (Applied Biosystem, Thermo Fisher Scientific) as recommended by the manufacturer. Briefly, 9.5 µL of the reaction mix, containing 5 µL of Fast Universal Master Mix, 3.5 µL of RNAse-free water, 0.5 µL of TaqMan^®^ Gene expression assay (specific for the following genes: Neural cell adhesion molecule 1 (Ncam1) for myoblast phenotype, Paired box 3 (Pax3) for myoblast proliferation, and apoptosis regulator (Bcl2)), and 0.5 µL cDNA (corresponding to 10 ng total RNA). The cDNA was amplified (in duplicate) at the following thermal protocol: 95 °C for 20 s (reverse transcriptase inactivation), 95 °C for 1 s, and 60 °C for 20 s (40 PCR cycles). The 18 S was used as the housekeeping gene. The mRNA expression levels were normalized against 18 S, and the relative mRNA expression levels were calculated using the formula 2^−∆∆Ct^.

### Quantification of soluble paracrine factors released from SF µgels

The amount of vascular endothelial growth factor (VEGF) secreted by the myoblasts was measured using a Bio-Plex Pro^TM^ Mouse Cytokine 9-plex immunoassay 96-well kit (Bio-Rad Laboratory, Hercules, CA, USA), according to the manufacturer’s instructions. Briefly, undiluted supernatants were transferred onto separate wells of a 96-well plate, then the plates were read on the BioPlex 200 system (Bio-Rad), powered by Luminex xMAP technology. Data were expressed as pg of VEGF per mL.

### Statistical analysis

Statistical analysis was carried out with GraphPad version 8 (GraphPad Software, La Jolla, CA, USA). Data were initially analyzed using the Shapiro-Wilk normality test. Comparisons between groups were performed with the one-way analysis of variance (ANOVA) and the multiple t-tests. Significance was retained when *p* < 0.05. Experiments were performed at least in triplicate.

## Results and discussion

In recent years, therapeutic strategies relying on cell encapsulation have been in the spotlight [51–53]. Despite the many efforts that have been devoted to improving current therapies, open challenges, including limited cell survival and passive cell displacement have hampered progress in this area. These limitations have fueled much interest in the design of delivery vehicles to enhance the viability and function of cells injected in situ [54, 55]. In this scenario, hydrogels have emerged as a powerful solution to these quests, as they provide convenient biophysical-chemical cues to cells for their survival and maintenance [56–58].

### Control of ultrasonication-induced SF gelation

**Fig. 2:**
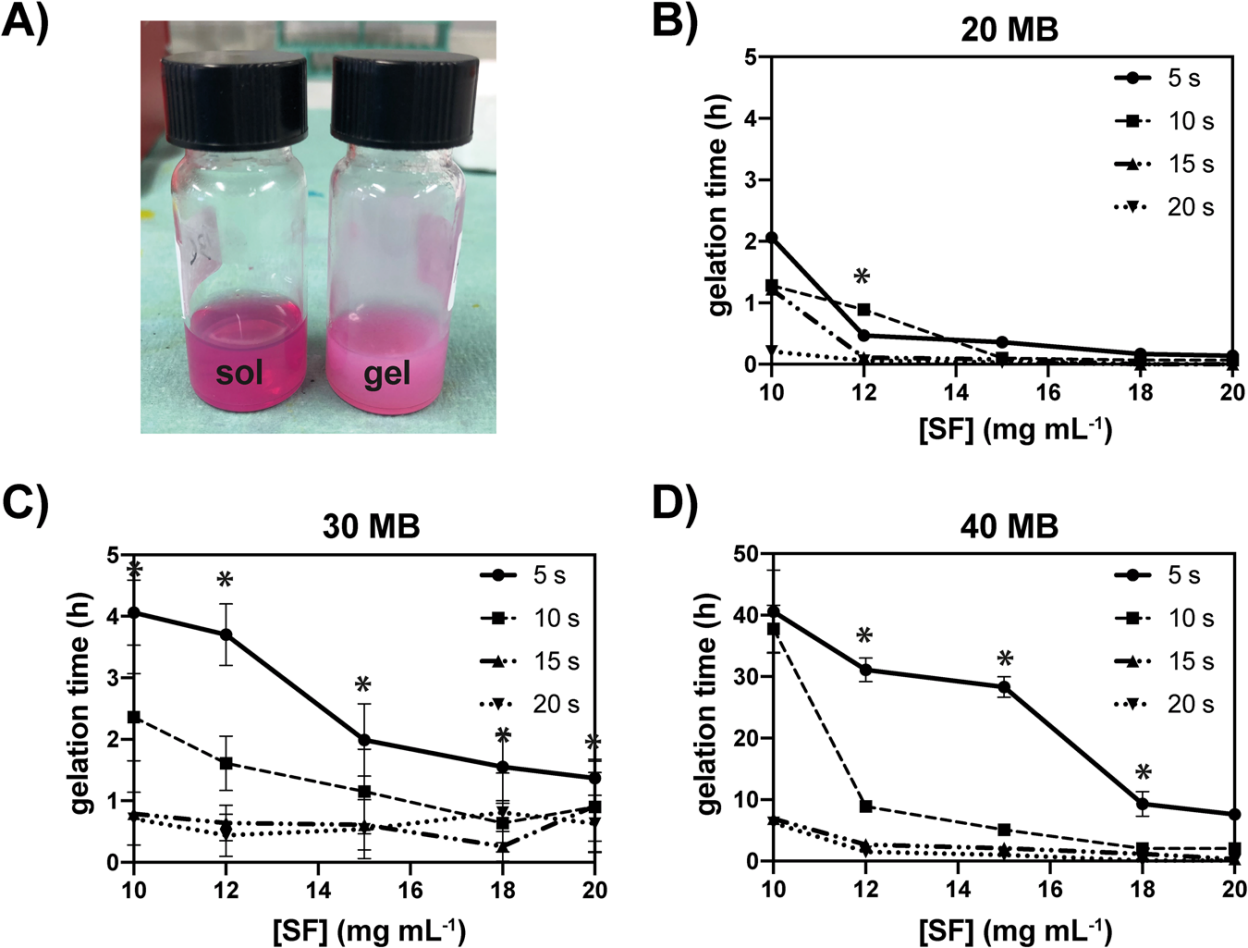
SF gelation time under various sonication conditions. **A** Representative SF solution and hydrogel prepared in DMEM. **B**–**D** Gelation time of SF solutions prepared in DMEM at different SF concentrations (from 10 to 20 mg mL^−1^) and boiling times (20, 30, and 40 MB). The different SF aqueous solutions were sonicated at 30% amplitude for different sonication times (5, 10, 15, and 20 s). Data are expressed as mean ± SD (*p < 0.05 among different sonication periods). The sol-gel transition was monitored by visual inspection of the samples

The kinetics of SF gelation was carefully characterized to develop a fabrication protocol for the production of µgels. In the present study, ultrasonication was used to trigger the sol-gel transition of the SF [44, 48]. Because the gelation time is a critical parameter for the production of hydrogels and the subsequent cell encapsulation, we investigated the effects of various sonication durations on the gelation kinetics of SF solutions prepared in different aqueous media (MilliQ water, PBS, and DMEM), at different SF concentrations (ranging from 10 to 20 mg mL^−1^), and the SF M_W_ tuned by controlling the boiling time (20, 30, and 40 min). Notably, the SF concentrations used in this study were much lower than those reported in the literature [44], and were specially selected to gain control over the gelation kinetics. The results are summarized in Fig. 2A. While keeping the power output constant at 30% amplitude, the SF gelation time invariably decreased with increased sonication time (Fig. 2). Different boiling times resulted in SF solutions with very different gelation kinetics. More in detail, when the 20 MB SF solution was employed, the gelation occurred within 2 h, when a 5 s sonication was applied. In comparison, the gelation time increased to 4 h or even 40 h when 30 MB and 40 MB SF solutions, respectively, were used (Fig. 2B–D). Furthermore, the higher the SF concentration, the shorter the gelation time.

Based on such results, we decided to use a 30 MB SF solution at a concentration of 15 mg mL^−1^, sonicated for 15 s at 30% amplitude for all further experiments. With this combination of parameters, the onset of the gelation starts after 1 h, providing enough time to conveniently mix the sonicated solution with cells, while SF is in the sol state, and to create micro size droplets in the emulsion. In this way the SF sets to create the microcapsules without the cells experiencing any photo- or chemical-based trigger that may be potentially harmful to them.

### SF µgels fabrication and characterization

**Fig. 3:**
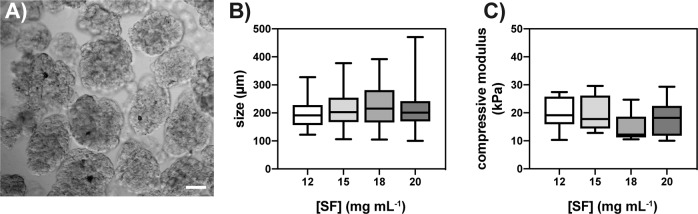
Morphological and mechanical characterization of SF µgels. **A** Digital picture of the 30 MB SF µgels (SF: 15 mg mL^−1^) imaged through an optical microscope. Scale bar = 50 µm. Morphological and mechanical characterization of SF µgels prepared in PBS at different SF concentrations: (**B**) size distribution; (**C**) compressive (elastic) moduli calculated through microindentation tests. Data are represented with inter-quartile range box plots and whiskers indicating the min and the max percentiles. Solid lines in the box plot indicate the median value of each dataset

Building on the control of gelation time, SF µgels were prepared via a batch emulsion procedure shown in Fig. S1. As reported in Fig. 3A, optical microscopy confirms that SF µgels were roughly spherical in shape, with an average diameter of ≈200 µm. This size is considered optimal for cell encapsulation and survival because very suited for the exchange of metabolites and gases in and out of the gels and for the secretion of biomolecules (e.g., paracrine factors) through the gel matrix [11, 59–61]. Small variations in the median value were observed among µgels prepared at different SF concentrations (Fig. 3B). This means that the concentration of SF, which modifies the viscosity of the aqueous phase, has a minor effect on the ultimate size of the µgels.

As shown by SEM images (Fig. S3A–B), SF µgels had a peculiar microstructure characterized by the presence of a dense network of nanosized silk fibrils (Ø ≈ 20 nm). The more compact morphology observed at the external surface of the µgels was probably due to a different self-assembly mechanism at the water-oil interface.

FTIR analyses allowed the identification of the secondary structure of the SF proteins (Fig. S3C–D). Specifically, the spectrum reported in Fig. S3D, obtained by self-deconvolution of the IR absorption peak of Amide I between 1700 and 1600 cm^−1^, showed the presence of β-sheets (≈50% of the area of Amide I band) typical of a highly crystalline and water-stable SF [34].

The compressive strength of the µgels was assessed by microindentation tests. Representative images of SF µgels captured during the tests are reported in Figs. S2A–B, and the results are summarized in Fig. 3C. The SF µgels showed compressive moduli ranging between 15 kPa and 25 kPa, with little influence of SF concentration on the compressive modulus. Overall, these values are significantly higher than those reported in the literature for other natural cell-encapsulating hydrogels made of collagen and gelatin [62, 63]. Of note, when SF gels were prepared at 12 and 15 mg mL^−1^, µgels showed a compressive modulus ≈2-fold greater than that of the macroscopic counterparts, tested in non-confined compression tests, and displayed compressive moduli ranging between 3 and 15 kPa (Fig. S5), in agreement with the previously published data [44]. These results hint that the mechanical properties of hydrogels are strongly influenced by their morphology and microstructure. We speculate that these differences might be ascribed to a stiffer peel present on the surface of the µgels. To support this hypothesis, both the inner core and the outer surface of a macroscopic cylindrical hydrogel were analyzed and compared to the SF µgels. As reported in Fig. S4B, there was a sharp difference between the stiffness of the inner core of the macroscopic hydrogel (compressive modulus ≈3 kPa) and the outer surface of the macrogels (compressive modulus ≈25 kPa), the latter being close to the one measured for the SF µgels (Figs. 3C, and S4B).

Such results suggest that the microstructuration of SF in the µgels leads to a significant improvement in their overall mechanical performances, providing a new mechanism to overcome the usually poor mechanical properties of protein-based hydrogels, one of their most critical shortcomings.

### Evaluation of SF sterilization upon sonication

Aseptic processing is the preferred way to ensure hydrogel sterility. As a matter of fact, if aseptic conditions are employed during material processing, the material itself can be considered sterile [64]. On the other hand, terminal sterilization via ethylene oxide or gamma radiation is not appropriate for cell-laden materials because it will be detrimental to cells. Furthermore, these methods dramatically affect the features and the behavior of the hydrogels, such that they may tamper with the gelation process or, if the polymer has already gelled, modify the surface and mechanical properties of the resulting hydrogel to some extent. Here, we evaluated if the ultrasonication process, used to speed up the gelation of the SF, could exert some degree of antimicrobial activity, providing a way to make the solution(s) sterile. To validate this hypothesis, the SF solutions were loaded with a high-titer suspension of *E. coli*, and the resulting mixture was sonicated for 10 s at 20 kHz and 30% amplitude. It is worth reminding that these conditions induce faster SF gelation. As shown in Fig. S5, no viable bacteria were detected on Agar plates after applying this process (Fig. S5B), demonstrating a complete bacteria killing upon ultrasonication (*E. coli* inoculum ≈ 10^7 ^mL^−1^). This is not surprising as ultrasonication has been extensively employed for cell disruption and disinfection purposes [65–67]. Overall, these results disclose ultrasonication as a reliable means of terminal sterilization of SF solutions in addition to being a smart way to trigger gelation eventually.

### Cells encapsulation and VEGF release

When designing hydrogels for cell encapsulation, it is essential to consider how the process used to encapsulate the cells can achieve high encapsulation efficiency, maintain high viability, and optimize the environment in which the cells are embedded. All three of these aspects were tested for the SF µgels.

**Fig. 4:**
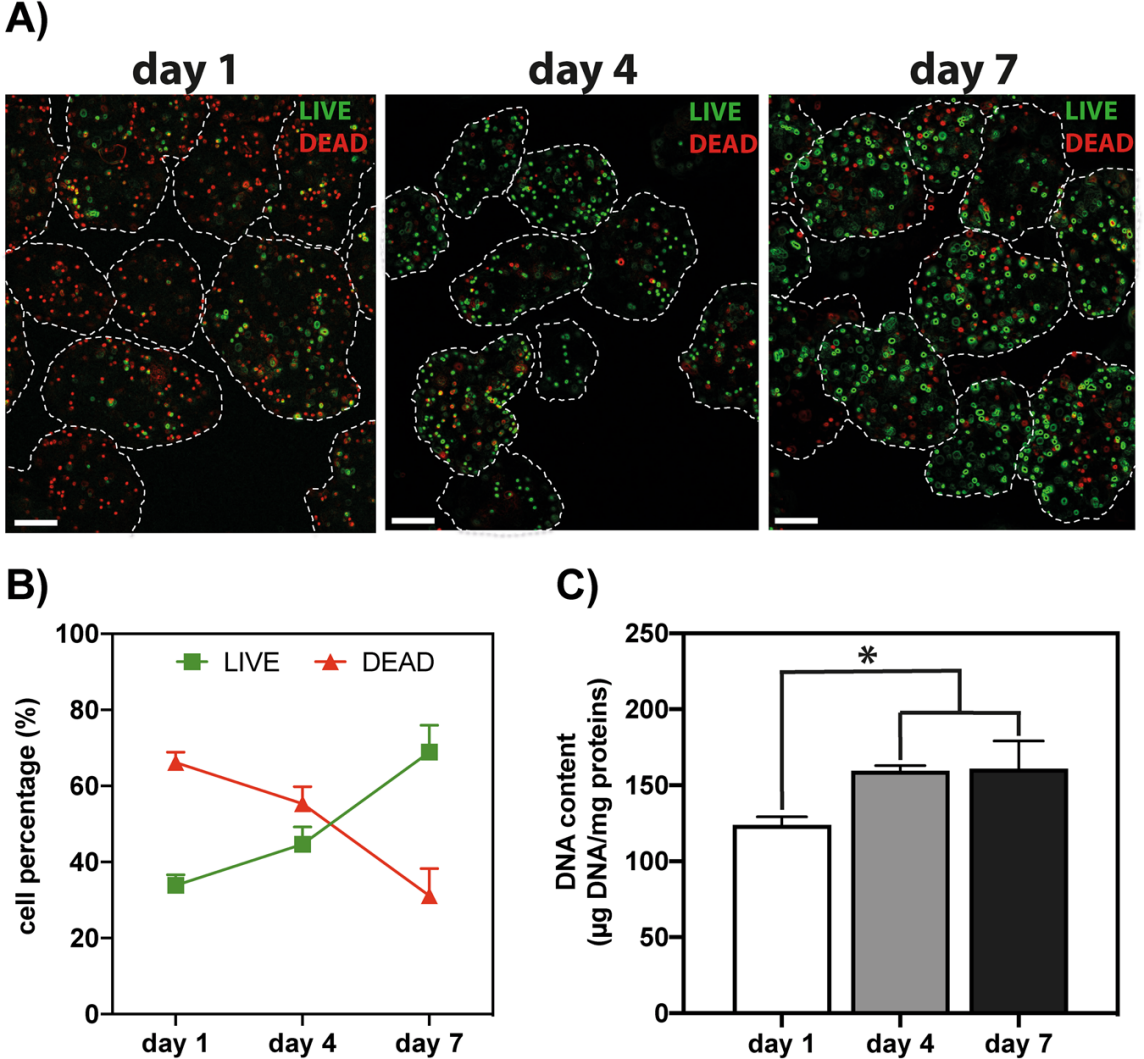
Biochemical characterization of L929 cell-encapsulating SF µgels. **A** Live/Dead fluorescence images of L929 cells encapsulated in 30 MB SF µgels and cultured for 1, 4, and 7 days (green: live; red: dead; scale bar: 100 µm; 40× magnification). **B** Quantification of cell viability of encapsulated L929 cells in SF µgels cultured for 1, 4, and 7 days post-cell encapsulation, expressed as the percentage (%) of live and dead cells. **C** Quantification of DNA content in L929-encapsulating SF µgels cultured for 1, 4, and 7 days. Data are expressed as µg DNA per mg of SF within the µgels

We first assessed whether the encapsulation process is cell-friendly. To this aim, fast-proliferating L929 cells were encapsulated within the SF µgels, and their viability was monitored over an extended time using the Live/Dead assay (Fig. 4). As expected, SF boasted excellent biocompatibility, and the number of viable cells embedded in the µgels steadily increased from ≈30% on day 1 to ≈70% on day 7 (Fig. 4B). The quantification of the DNA content in the µgels corroborated these findings (Fig. 4C). Indeed, a significant increase in the total cell DNA content was found in SF µgels after 4 and 7 days of culture with respect to day 1 (*p* < 0.05).

Overall, these results demonstrated the reliability of our protocol to fabricate cell-encapsulating SF µgels and that SF µgels kept cells alive once encapsulated. The gelation time chosen was sufficiently long to allow proper cell loading and short enough to preserve cell viability during fabrication. The control on process parameters using the data presented in Fig. 2 allows for an increase or decrease this time window to accommodate experimental needs, such as fabrication time or cell sensitivity.

It is worthy of note that the design of cell-encapsulating µgels should also deal with matrix porosity and degradability. The porosity determines the cut-off size (M_W_) for molecules to diffuse in and out of the hydrogel. In this work, the selective permeability of SF µgels to relevant biomacromolecules, such as growth factors and antibodies, was also assessed.

**Fig. 5:**
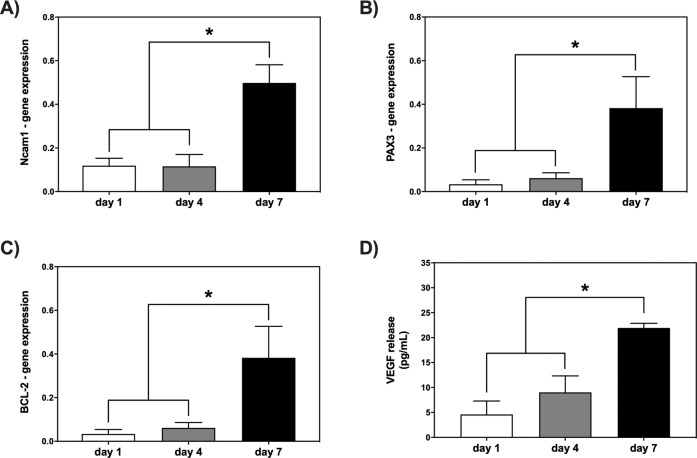
Increased expression levels of Ncam1, Pax3, BCL-2 genes, and VEGF paracrine factor in myoblasts on day 7 after cell encapsulation. Quantitative real-time PCR analysis of Ncam1 (**A**), Pax3 (**B**), and Bcl-2 (**C**) genes in total RNA extracted from myoblasts on days 1 (white bars), 4 (grey bars), and 7 (black bars) post-cell encapsulation (*n* = 4 per group). Relative expression data are presented as mean ± SD of 2^−∆∆Ct^ values normalized against endogenous control 18 S (*p < 0.05). **D** VEGF levels (pg mL^−1^) quantified into the supernatant on days 1, 4, and 7 after cell encapsulation (*n* = 4 per group) were measured by multiplex immunoassay. Data are expressed as mean ± SD for each sample group (*p < 0.05)

To do so, extremely sensitive primary myoblasts were encapsulated into SF µgels, and their proliferation and the secretion of VEGF (40 kDa) were evaluated over time (1, 3 and 7 days post cell encapsulation). We investigated by qRT-PCR three regulatory messengers that are the targets of myoblast cell survival [68], namely Ncam1 and Pax3 transcripts, that are specific myoblast proliferation markers [69], and Bcl2, a known anti-apoptotic regulator [70]. As depicted in Fig. 5A, B, there was a significant increase (*p* < 0.05) in the expression levels of Ncam1 and Pax3 on day 7, if compared with days 1 and 3. These results show that the viability and proliferation of myoblasts were increasingly better as the myoblasts were cultured into the SF µgels. Of note, overall good behavior was associated with a concomitant increased expression of the Bcl2 gene (Fig. 5C; *p* < 0.05). Altogether, these findings suggest that the microenvironment inside the SF µgels was suitable for the maintenance of the myoblast cell functions.

Along with myoblast cell survival, the release of VEGF was quantified into the supernatant on 1, 4, and 7 days post-cell encapsulation. Figure 5D shows that the VEGF levels slightly increased from day 1 (4.5 pg mL^−1^) to day 4 (9 pg mL^−1^) and significantly rose further on day 7 (22 pg mL^−1^). Such results are very interesting, especially considering that VEGF is a potent angiogenic growth factor also known to inhibit myoblast apoptosis [71]. This implies that some signaling biomolecules and metabolites can freely diffuse out of the µgel. On the other hand, other macromolecules (M_W_ ≥ 150 kDa), such as IgG antibodies, cannot enter the µgels (Fig. S6) but stick on their surface. Indeed, despite the IgGs are few nm in size, their entry is dependent also on environmental conditions such as the pH and salt concentration so that the interaction between microgels and proteins may be affected. Overall, such results suggest that the SF µgels possess a selective permeability to biomacromolecules.

Another key aspect to be considered is the stability of the hydrogels used for cell encapsulation. Interestingly, despite the small size and the high cell loading, no release of cells from µgels was observed over one week of in vitro cell culture, and the µgels were able to preserve their shape and size, meaning that SF µgels are stable over this timeframe. This observation is in good agreement with previous findings on macroscopic SF hydrogel constructs, which have shown to take 2-to-6 months to lose stability in vivo, that is, to degrade [72, 73]. In this context, understanding and predicting how SF degradation may occur in the presence of human proteases is of great importance, especially for regenerative medicine purposes. With this in mind, we employed the integrated feature-based server iProt-Sub to identify in silico which of the different human extracellular proteases recognize specific cleavage sites within the SF protein, namely SF heavy chain, light chain, and P25 protein sequences [74, 75]. It is worth noting that this server took into account the restricted accessibility to proteases due to the 3D structure of the target protein, and not just the presence of recognition sequences for specific enzymes in the primary SF structure. We found that matrix metalloproteinases (MMPs) and serine proteases have some cleavage sites within the SF protein (Table S1), according to previously published data [76]. These findings may help address the SF degradation in different tissues and applications.

## Conclusions

In this study, we proposed a simple method for the fabrication of cell-encapsulating SF µgels. The strategy herein presented relies on the production of microdroplets of SF solution in a batch emulsion. The sol-gel transition was triggered by sonication of the silk solution before mixing them with cells, that will not experience non-physiological conditions during the fabrication of the µgels. We precisely characterized the time window of the sol-gel transition, so that it can be controlled according to the experimental needs of different applications. In our study, we selected the most relevant parameters that allowed gelation in 1 h, a time that was long enough to allow cell loading and short enough to preserve cell viability. The SF µgels produced with our method had a narrow size distribution (Ø ≈ 200 µm) and improved mechanical properties. This made the µgels suitable for handling without releasing encapsulated cells. The selective porosity of SF µgels allowed the exchange of metabolites, and gases to keep the encapsulated cells alive and proliferating, and the release of biomacromolecules such as VEGF (M_W_ = 40 kDa) through the gel network, while restricting the entry of other molecules such as IgG. These results lay the groundwork for further uses of these µgels in vitro and in vivo. In perspective, these SF µgels have the potential for advanced therapies in many fields, including neuronal and muscular disorders.

## Supplementary information

Supplementary Information
