# Supplementary material for: Silk fibroin microgels as a platform for cell microencapsulation
Source: J Mater Sci Mater Med. 2022 Dec 31;34(1):3. doi: 10.1007/s10856-022-06706-y (PMC9805413; doi:10.1007/s10856-022-06706-y)
Supplement: Supplementary file 1 — Supplementary Information [file 10856_2022_6706_MOESM1_ESM.docx]

Supplementary Information

**Title:**

Silk fibroin microgels as a platform for cell microencapsulation

**Authors List:**

Nina Bono^1^*, Giulio Saroglia^1,2^, Stefania Marcuzzo^3^, Eleonora Giagnorio^3^, Giuseppe Lauria^4,5^, Elena Rosini^6^, Luigi De Nardo^1^, Athanassia Athanassiou^2^, Gabriele Candiani^1^ and Giovanni Perotto^2^*

^1^ Dept. of Chemistry, Materials and Chemical Engineering "Giulio Natta", Politecnico di Milano, Via Mancinelli 7, 20131 Milan, Italy

^2^ Smart Materials, Istituto Italiano di Tecnologia, Via Morego 30, 16163, Genova, Italy

^3^ Neurology IV-Neuroimmunology and Neuromuscular Diseases Unit, Fondazione IRCCS Istituto Neurologico Carlo Besta, Via Celoria 11, 20133, Milan, Italy

^4^ Dept. of Clinical Neurosciences, Fondazione IRCCS Istituto Neurologico Carlo Besta, Via Celoria 11, 20133, Milan, Italy

^5^ Dept. of Medical Biotechnology and Translational Medicine, University of Milan, Milan, Italy, Via Vanvitelli 32, 20133, Milan, Italy

^6^ The Protein Factory 2.0, Dept. of Biotechnology and Life Sciences, University of Insubria, Via J.H. Dunant 3, 21100, Varese, Italy

**Corresponding Authors**

Nina Bono^1^*

^1^ Dept. of Chemistry, Materials and Chemical Engineering "Giulio Natta", Politecnico di Milano, Via Mancinelli 7, 20131, Milan, Italy

tel.: +39-02-2399-3045; e-mail: [nina.bono@polimi.it](mailto:nina.bono@polimi.it) ; ORCID: 0000-0002-6891-9879

Giovanni Perotto^2^*

^2^ Smart Materials, Istituto Italiano di Tecnologia, Via Morego 30, 16163, Genova, Italy

tel.: +39-010-2896-773; e-mail: [giovanni.perotto@iit.it](mailto:giovanni.perotto@iit.it); ORCID: 0000-0001-8467-8748

**Table of contents**

[S1. Unconfined compression tests on SF macrogels 4](#_Toc108459979)

[S2. Evaluation of SF permeability to macromolecules 4](#_Toc108459980)

[Figure S1: Schematic representation of SF µgels fabrication process. 5](#_Toc108459981)

[Figure S2: Mechanical characterization of SF µgels via microindentation tests. 6](#_Toc108459982)

[Figure S3: Morphological and physical-chemical characterization of SF µgels 7](#_Toc108459983)

[Figure S4: Mechanical characterization of SF macrogels. 8](#_Toc108459984)

[Figure S5: Qualitative observation of microbial decontamination in SF-bacteria mixture. 9](#_Toc108459985)

[Figure S6: Permeability of SF µgels to macromolecules. 10](#_Toc108459986)

[Table S1: *In silico* prediction of cleavage sites within SF 11](#_Toc108459987)

[References 12](#_Toc108459988)

# **Unconfined compression tests on SF macrogels**

To measure the stiffness of SF macrogels, macroscopic cylindrical hydrogels were characterized in unconfined compression tests using an Instron machine (Instron 3365, Norwood, Massachusetts, USA) equipped with a 2.5 kN load cell. Briefly, macrogels (dimensions: 10 mm × 10 mm × 16 mm) were prepared by pouring the sonicated SF solutions into the PDMS molds, and kept at 37 °C to allow gelation. Compression tests were performed in wet conditions at r.t. a compression rate of 10 mm min^-1^ (limit: 90 % compression). The compressive modulus values of SF macrogels (n = 3 per condition) were calculated from the slope of the linear stress-strain plot (comprised between 5 and 30 % of the strain).[1]

# **Evaluation of the permeability of SF µgels to macromolecules**

The permeability of SF µgels was preliminarily evaluated by confocal microscopy. Briefly, following fabrication, the SF µgels were incubated for 24 hrs with 10 mL of FITC-donkey anti-mouse immunoglobulin G (IgG; 0.1 mg mL^-1^; M_W_ = 160 kDa; Jackson ImmunoResearch Europe, Ely, UK) in PBS. After a 24 hr-incubation, confocal images were acquired using a Nikon A1 confocal microscope equipped with a 488 nm Argon laser (excitation) and collecting light through a 525/50 (green) bandpass filter to detect FITC fluorescence.

# **Figure S1:** **Schematic representation production process of SF µgels.**

SF solutions were prepared at different SF concentrations and in different aqueous solutions (MilliQ, PBS, or DMEM) and were subjected to different sonication parameters (time and amplitude). Afterward, SF µgels were prepared through the batch emulsion procedure. Image created with BioRendem.com


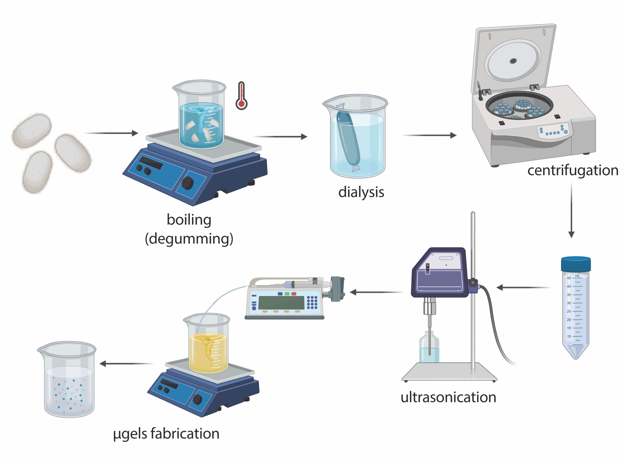


# **Figure S2: Mechanical characterization of SF µgels via microindentation tests.**

**A-B)** Representative image of an SF µgel during microindentation tests. **C-D)** Typical load-displacement profiles obtained for SF µgels are shown in **A)** and **B)**, respectively.


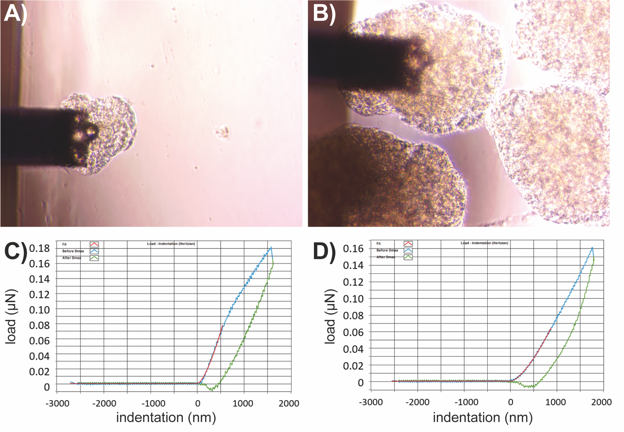


# **Figure S3: Morphological and physical-chemical characterization of SF µgels**

SEM micrographs of SF µgels (SF: 15 mg mL^-1^): **A)** whole µgel capsule; **B)** internal structure of an SF µgel; **C)** FTIR spectrum, and **D)** fitting of the Amide I band of the SF µgels.


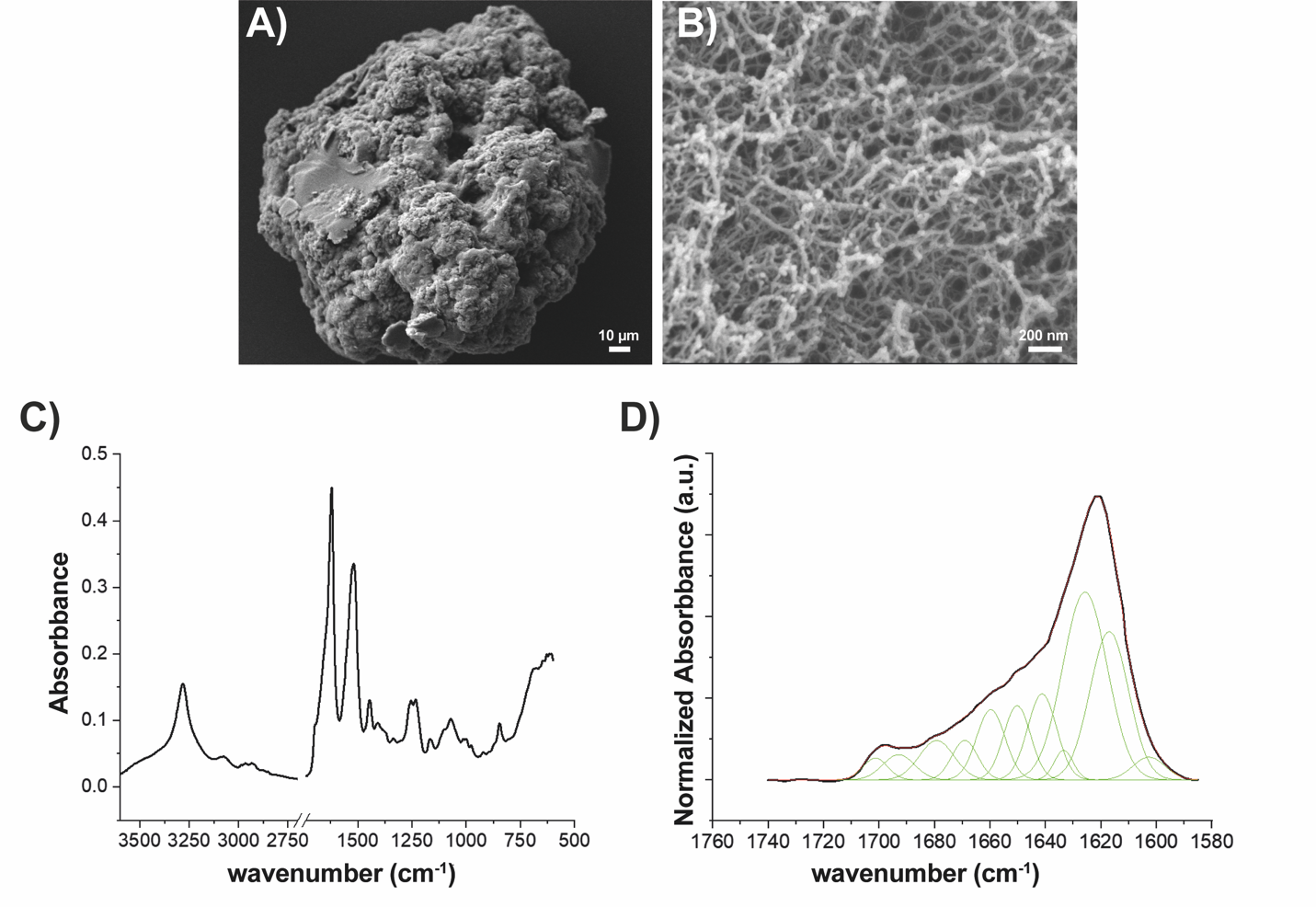


# **Figure S4: Mechanical characterization of SF macrogels.**

**A)** Compressive moduli measured by non-confined compression on macroscopic hydrogels (10 mm diameter × 16 mm height) prepared in PBS at different SF concentrations. Data are represented with inter-quartile range box plots and whiskers indicating the min and max percentiles. Solid lines in the box plot indicate the median value of each dataset. **B)** Comparison between the compressive moduli of a representative SF formulation measured by microindentation tests on the inner core and the outer rim (surface) of a macroscopic hydrogel.


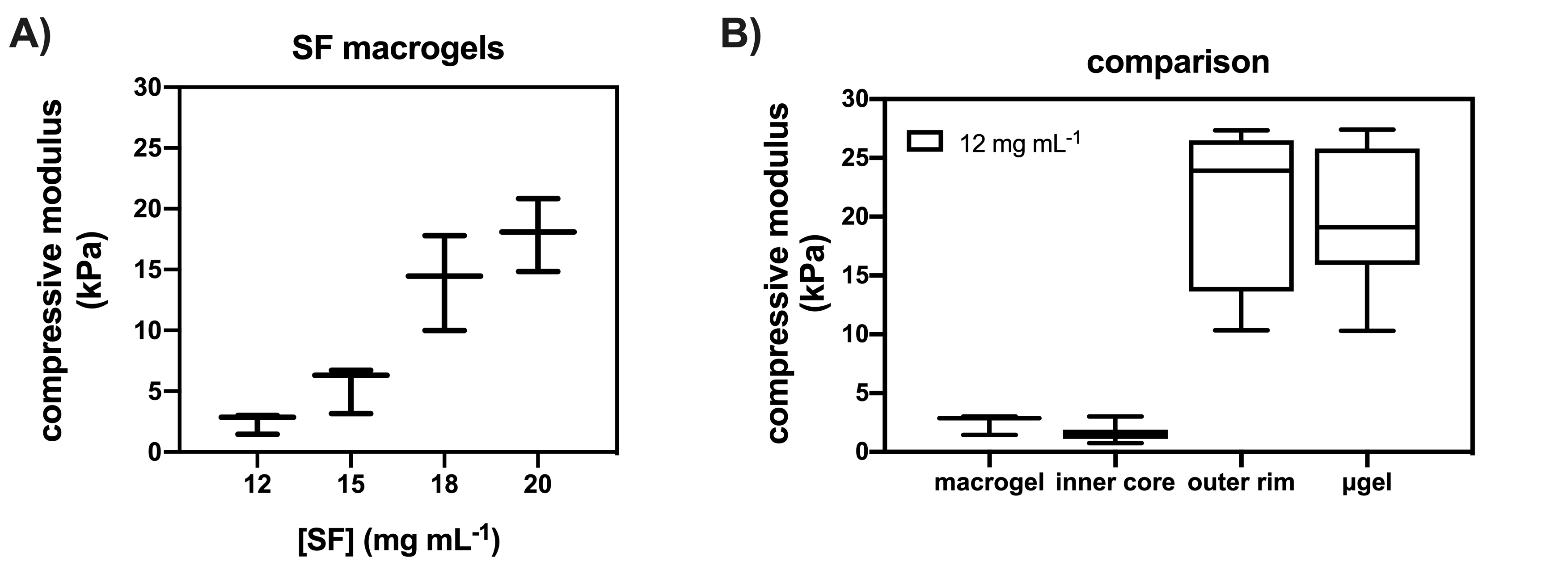


# **Figure S5: Qualitative observation of microbial decontamination in SF-bacteria mixture.**

Qualitative observation of microbial decontamination of A) non-sonicated and B) sonicated SF solutions after plating onto agar plates. No viable bacteria were found in sonicated SF solution.


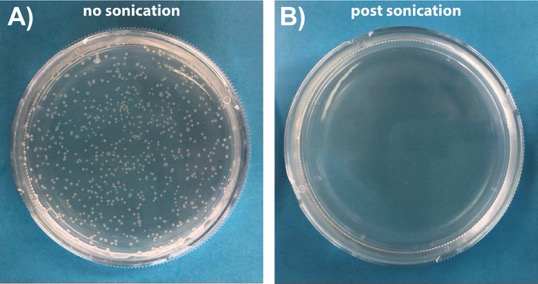


# **Figure S6: Permeability of SF µgels to macromolecules.**

Assessment of the permeability of SF µgels to FITC-donkey anti mouse IgG (M_W_ = 160 kDa).


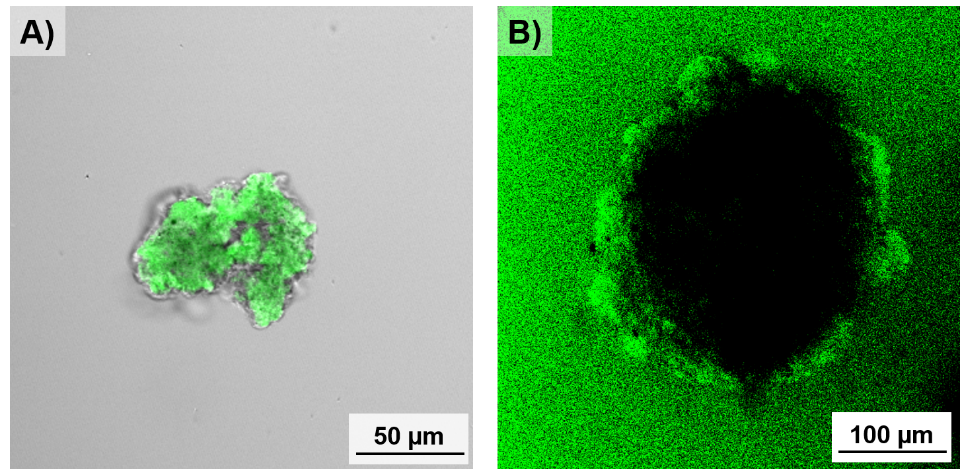


# **Table S1: *In silico* prediction of cleavage sites within SF**

Cleavage sites of extracellular human proteases within the silk fibroin heavy chain (HC), light chain (LC), and P25 proteins, as predicted by the integrated feature-based server iProt-Sub (<http://iprot-sub.erc.monash.edu.au/home.html>).

|  | **# cleavage sites** | | |
| --- | --- | --- | --- |
|  | **HC** | **LC** | **P25** |
| **Metallo protease family** |  |  |  |
| Matrix metallopeptidase 1 (collagenase 1) |  |  | 1 |
| Matrix metallopeptidase 2  (gelatinase A) |  |  | 5 |
| Matrix metallopeptidase 3  (stromelysin 1) |  |  | 3 |
| Matrix metallopeptidase 7  (matrilysin) |  |  | 1 |
| Matrix metallopeptidase 8  (collagenase 2) |  |  | 2 |
| Matrix metallopeptidase 9  (gelatinase B) |  |  | 4 |
| Matrix metallopeptidase 12  (macrophage elastase) |  |  | 4 |
| Matrix metallopeptidase 13  (collagenase 3) |  |  | 2 |
| **Serine protease family** |  |  |  |
| Elastase 2 | 26 | 1 | 4 |
| Granzyme A | 2 | 2 | 4 |
| Granzyme B | 2 | 2 | 10 |
| Kallikrein Peptidase 4 | 4 | 1 | 4 |
| Kallikrein Peptidase 5 | 5 | 3 | 1 |
| Plasmin | 3 | 4 | 2 |

# **References**

1. Caldwell AS, Campbell GT, T Shekiro KM, Anseth KS, Caldwell AS, Campbell GT, et al. Clickable Microgel Scaffolds as Platforms for 3D Cell Encapsulation. Adv Healthc Mater [Internet]. John Wiley & Sons, Ltd; 2017 [cited 2022 Aug 22];6:1700254. Available from: https://onlinelibrary.wiley.com/doi/full/10.1002/adhm.201700254
